# Cloning and Functional Characterization of the Maize (*Zea mays* L.) Carotenoid Epsilon Hydroxylase Gene

**DOI:** 10.1371/journal.pone.0128758

**Published:** 2015-06-01

**Authors:** Shu Chang, Judit Berman, Yanmin Sheng, Yingdian Wang, Teresa Capell, Lianxuan Shi, Xiuzhen Ni, Gerhard Sandmann, Paul Christou, Changfu Zhu

**Affiliations:** 1 School of Life Sciences, Changchun Normal University, Changchun, 130032, China; 2 Departament de Producció Vegetal i Ciència Forestal, Universitat de Lleida-Agrotecnio Center, Lleida, 25198, Spain; 3 School of Life Sciences, Beijing Normal University, Beijing, 100875, China; 4 School of Life Sciences, Northeast Normal University, Changchun, 130024, China; 5 Biosynthesis Group, Molecular Biosciences, Goethe University Frankfurt, D-60438, Frankfurt, Germany; 6 Institució Catalana de Recerca i Estudis Avancats, Barcelona, 08010, Spain; Washington State University, UNITED STATES

## Abstract

The assignment of functions to genes in the carotenoid biosynthesis pathway is necessary to understand how the pathway is regulated and to obtain the basic information required for metabolic engineering. Few carotenoid ε-hydroxylases have been functionally characterized in plants although this would provide insight into the hydroxylation steps in the pathway. We therefore isolated mRNA from the endosperm of maize (*Zea mays* L., inbred line B73) and cloned a full-length cDNA encoding CYP97C19, a putative heme-containing carotenoid ε hydroxylase and member of the cytochrome P450 family. The corresponding *CYP97C19* genomic locus on chromosome 1 was found to comprise a single-copy gene with nine introns. We expressed *CYP97C19* cDNA under the control of the constitutive CaMV 35S promoter in the *Arabidopsis thaliana lut1* knockout mutant, which lacks a functional *CYP97C1 (LUT1*) gene. The analysis of carotenoid levels and composition showed that lutein accumulated to high levels in the rosette leaves of the transgenic lines but not in the untransformed *lut1* mutants. These results allowed the unambiguous functional annotation of maize CYP97C19 as an enzyme with strong zeinoxanthin ε-ring hydroxylation activity.

## Introduction

**Fig 1:**
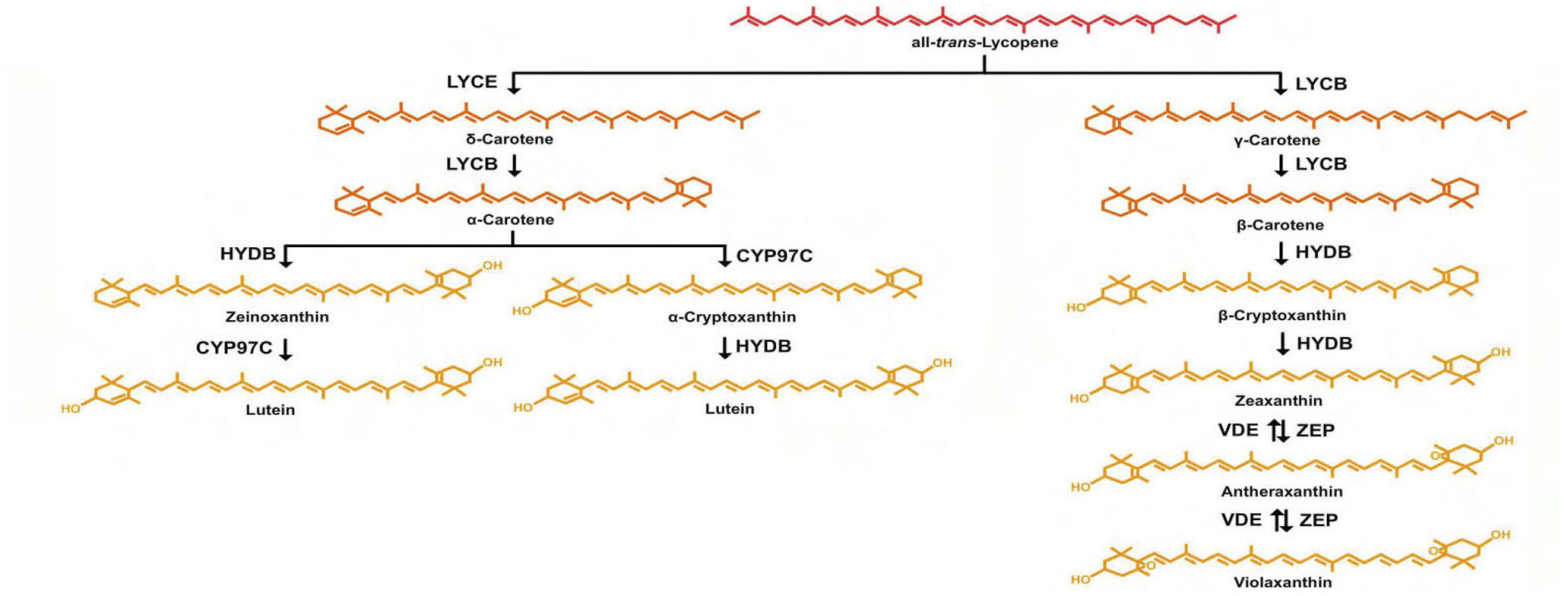
Xanthophyll biosynthesis pathway in plants. Abbreviations: CYP97C, heme-containing cytochrome P450 carotene ε-ring hydroxylase; HYDB, *β*-carotene hydroxylase (non-heme di-iron *β*-carotene hydroxylase (BCH) and heme-containing cytochrome P450 β-ring hydroxylases CYP97A and CYP97B); LYCB, lycopene β-cyclase; LYCE, lycopene ε-cyclase; VDE, violaxanthin de-epoxidase; ZEP, zeaxanthin epoxidase.

Carotenoids play a fundamental role in human and animal nutrition. Lutein, zeaxanthin and lycopene from plant sources act as antioxidants and protect against diseases such as cancer, whereas others such as β-carotene, β-cryptoxanthin and α-carotene are precursors of vitamin A and retinoid compounds, which are essential for vision, a strong immune system and normal development [1–4]. Carotenoids in plants are synthesized in the plastids [5–6]. The first committed step in carotenoid synthesis is the condensation of two molecules of geranylgeranyl diphosphate (GGPP) by phytoene synthase (PSY) to produce phytoene, which is then converted into all-*trans*-lycopene via four desaturation and isomerization steps. Lycopene is a branching point, leading to either β-carotene via γ-carotene or to α-carotene via ε-carotene. The subsequent oxygenation of α-carotene by ε-ring hydroxylases yields lutein whereas the oxygenation of β-carotene by β-ring hydroxylases yields zeaxanthin (Fig 1).

Two pairs of duplicated carotenoid hydroxylases have been identified in *Arabidopsis thaliana* [7–9], rice [10] and tomato [11]. In *A*. *thaliana*, two genes encode non-heme di-iron β-carotene hydroxylases (BCH1 and BCH2) and two genes encode the heme-containing CYP-type cytochrome P450 hydroxylases carotenoid β-hydroxylase CYP97A3 and carotenoid ε-hydroxylase CYP97C1 [7–9,12,13](. The BCH-type enzymes are primarily responsible for the hydroxylation of β,β-carotenoids (such as β-carotene) whereas the CYP-type enzymes are primarily responsible for the hydroxylation of ε,β-carotenoids (such as α-carotene) but they overlap in activity, most notably by contributing to the hydroxylation of the β-ring of α-carotene [9].

Non-heme β-carotene hydroxylase genes from plants and bacteria have been cloned and functionally characterized [5,14,15]. Rice *CYP97C2* and *CYP97A4*, the orthologs of *A*. *thaliana CYP97C1* and *CYP97A3*, have been isolated and characterized by *in vitro* functional complementation in *Escherichia coli* [10]. Rice CYP97A4 acts on the β-ring of both α-carotene and β-carotene, but is inactive towards the ε-ring of α-carotene. Conversely, rice CYP97C2 shows high activity towards the ε-ring and moderate activity toward the β-ring of α-carotene, but minimal activity toward the β-rings of β-carotene in the *E*. *coli* functional complementation system [10]. The analysis of rice *cyp97a4* mutants showed that the carotenoid β-ring hydroxylase CYP97A4 plays an important role in α-carotene hydroxylation [16]. The tomato *CYP97C11* and *CYP97A29* genes have been characterized in transgenic tomato plants [11]. The constitutive overexpression of the tomato carotenoid ε-hydroxylase *CYP97C11* in transgenic tobacco significantly increased the amount of lutein in the leaves and helped to alleviate photo-inhibition and photo-oxidation induced by chilling stress [17]. The cytochrome P450 type β-carotene hydroxylase CYP175 is exclusively present in the thermostable bacterium *Thermus thermophilus* HB27 and the yeast *Xanthophyllomyces dendrorhous*, which converts β-carotene to zeaxanthin [18,19]. Recently, the cytochrome P450 type enzyme PuCHY1 (CYP97B subfamily) from the red alga *Porphyra umbilicalis* was also identified as a functional β-carotene hydroxylase [20]. In contrast to the studies presented above, only three carotenoid ε-hydroxylases have been functionally characterized thus far, from *A*. *thaliana*, rice and tomato [7,9–11].

The functional annotation of genes in the carotenoid biosynthesis pathway is important to understand pathway regulation and to generate predictive data for metabolic engineering. The maize *CYP97C19* gene encodes a P450-type carotenoid hydroxylase and appears structurally orthologous to *A*. *thaliana CYP97C1*, but a comprehensive functional characterization has not been reported. Here we describe the isolation of the maize *CYP97C19* gene, its structural characterization and the functional analysis of the corresponding enzyme in transgenic *A*. *thaliana* plants.

## Materials and Methods

### Plant materials

Maize plants (*Zea mays* L. cv B73) were grown in the greenhouse and growth chamber at 28/20°C day/night temperature with a 10-h photoperiod and 60–90% relative humidity for the first 50 days, followed by maintenance at 21/18°C day/night temperature with a 16-h photoperiod thereafter. Plants were self-pollinated to obtain seeds. Mature leaf and endosperm tissues were frozen rapidly in liquid nitrogen and stored at -80°C.

*Arabidopsis thaliana* control plants, either wild-type *A*. *thaliana* Col-0 or the *lut1* mutant [7], and transgenic plants derived from them, were grown in a growth chamber or greenhouse with a 16-h photoperiod at 23°C. Harvested dry *A*. *thaliana* seeds were stored for 2 weeks at 4°C before planting in soil or on agar plates for selection.

### Nucleic acid isolation and cDNA synthesis

Genomic DNA was extracted from leaf tissue as described by Edwards et al. [21]. Total RNA was isolated using the RNeasy Plant Mini Kit (Qiagen, Valencia, CA, USA) and DNA was removed with DNase I (RNase-free DNase Set, Qiagen). Total RNA was quantified using a Nanodrop 1000 spectrophotometer (Thermo Scientific, Vernon Hills, Illinois, USA), and 2 μg total RNA was used as template for first strand cDNA synthesis with Ominiscript reverse transcriptase (Qiagen) in a 20-μl total reaction volume, following the manufacturer’s recommendations.

### Cloning and sequencing of the putative maize *CYP97C* cDNA

The rice *CYP97C2* cDNA (GenBank: AK065689) was used as a query to search the maize expressed sequence tag (EST) database, and matches were used to design primers for full-length cDNA cloning. EST sequences (GenBank: CF244398 and CF245241) from inbred line B73 were found with high sequence identity to the ends of the rice *CYP97C2* cDNA. The full-length cDNA amplified using 1 μl cDNA prepared as above from the endosperm of maize inbred line B73 25 days after pollination (DAP), primers 5′-CAC ACG GCG ATG CCT GCC ACG GTC TTC-3′ and 5′-TCT ATT TCG ATT CGC TCA GCG CTA ACT C-3′, and the GoTaq DNA Polymerase Kit (Promega, Madison, WI, USA) in a 50-μl reaction. The samples were heated to 95°C for 3 min, followed by 30 cycles at 94°C for 45 s, 60°C for 45 s and 72°C for 2 min. After the last amplification cycle, the samples were incubated at 72°C for 10 min. The products were purified from a 0.8% w/v agarose gel using the Geneclean II Kit (BIO 101 Systems, Solon, OH, USA) and cloned in the PCR II TOPO vector (TA Cloning Kit, Invitrogen, Carlsbad, CA, USA) for sequencing using the Big Dye Terminator v3.1 Cycle Sequencing Kit on a 3130x1 Genetic Analyzer (Applied Biosystems, Foster City, CA, USA).

### Bioinformatic analysis

The Maize Genetics and Genomic Database (MaizeGDB, http://www.maizegdb.org/), the GRAMENE database (http://www.gramene.org/) and GenBank (http://blast.ncbi.nlm.nih.gov/Blast.cgi) were searched for homologous sequences using BLAST, and multiple sequences were aligned using ClustalW2 (http://www.ebi.ac.uk/Tools/msa/clustalw2/). Protein sequences were screened for chloroplast signal peptides using the ChloroP 1.1 server at http://www.cbs.dtu.dk/services/ChloroP/ [22]. Gene structures were predicted by aligning mRNA to genomic DNA using Spidey (http://www.ncbi.nlm.nih.gov/spidey/).

### Construction of maize *CYP97C* gene expression vector for *A*. *thaliana*

Gene-specific primers, with a *Nco*I restriction site (underlined) in the forward primer 5′-CCA TGG ATT AGA TGC CTG CCA CGG TCT TCG CCT CC-3′ and a *Bst*EII restriction site (underlined) in the reverse primer 5′-GGT CAC CTA TTT CGA TTC GCT CAG CGC TAA CT-3′ were used to amplify the full-length maize *CYP97C19* coding sequence, which was then inserted into binary vector pCAMBIA1302 linearized with the same enzymes to yield pCAMBIA-ZmCYP97C19.

### Transformation and selection of *A*. *thaliana*

The pCAMBIA-ZmCYP97C19 plasmids were introduced into *Agrobacterium tumefaciens* strain GV3101 by electroporation [23] and the recombinant bacteria were grown at 28°C overnight before the *A*. *thaliana lut1* mutant was transformed using the floral dip method [24]. Axenic cultures for the *A*. *thaliana* seeds were wetted with 75% ethanol for 1 min, washed once with sterile water, surface sterilized with a 50% bleach (2.625% sodium hypochlorite) containing 0.05% Tween-20 for 10 min, and rinsed with sterile water five times. *A*. *thaliana* T1 seeds obtained after floral dip transformation were selected on 0.7% agar plates containing half-strength Murashige and Skoog (MS) medium [25] containing 1% sucrose and supplemented with 50 mg/l hygromycin B (Roche, Mannheim, Germany) for 10 days in growth chamber, before transfer to standard horticultural soil in the greenhouse. T2 seeds were harvested and germinated T2 seedlings were selected on half-strength MS medium containing 1% sucrose, 0.7% agar and 50 mg/l hygromycin B for 7 days in a growth chamber. Five hygromycin-resistant plants were transferred to individual glass pots (7 cm diameter x 11 cm) filled with MS medium containing 2% sucrose and 0.7% agar for 2 weeks in the growth chamber. *A*. *thaliana* (Col-0 and *lut1* mutant) plants were cultured on the same MS medium without hygromycin B as controls. For each line and control, the rosette leaves from at least 50 plants were pooled in three biological replicates for HPLC analysis, as well as DNA and RNA extraction.

### DNA and RNA analyses

Leaf genomic DNA (10 μg for *A*. *thaliana*) was digested with appropriate restriction enzymes and fractionated by 0.8% (w/v) agarose gel electrophoresis before blotting onto a positively-charged nylon membrane (Roche Diagnostics GmbH, Mannheim, Germany) according to the manufacturer’s instructions. Nucleic acids were fixed by UV crosslinking. The transferred DNA fragments were hybridized with appropriate digoxigenin-labeled probes at 42°C overnight using DIG Easy Hyb buffer (Roche). The membrane was washed twice for 10 min in 2x SSC, 0.1% SDS at room temperature, twice for 30 min in 0.5x SSC, 0.1% SDS at 68°C, once for 20 min in 0.2x SSC, 0.1% SDS at 68°C and then once for 10 min in 0.1x SSC, 0.1% SDS at 68°C. After immunological detection with anti-DIG-AP (Roche) chemiluminescence generated by disodium 3-(4-methoxyspiro(1,2-dioxetane-3,2′-(5′-chloro)tricyclo[3.3.1.1^3,7^]decan)-4-yl)phenyl phosphate (CSPD) (Roche) was detected on Kodak BioMax light film (Sigma-Aldrich, St. Louis, USA) according to the manufacturer’s instructions. The 1228-bp maize *CYP97C19* probe for DNA blot analysis was prepared by PCR under the conditions described above using primers 5′-GTC TCC GAG TTC CTC TTC GGC TCC GGC T-3′ and 5′-CTA TTT CGA TTC GCT CAG CGC TAA CTC A-3′.

Total RNA (20 μg) extracted from *A*. *thaliana* leaves was fractionated on a denaturing 1.2% (w/v) agarose gel containing formaldehyde prior to blotting. The membrane was probed with digoxigenin-labeled partial cDNAs prepared as above using the PCR-DIG Probe Synthesis Kit (Roche), with hybridization carried out at 50°C overnight using DIG Easy Hyb buffer and the same probe as described above. Washing, immunological detection and CSPD chemiluminescence were also carried out as described above.

### Carotenoid extraction and quantification

Carotenoids were extracted from *A*. *thaliana* freeze-dried leaves by heating in methanol containing 6% KOH for 20 min at 60°C. The extract was partitioned into 10% ether in petroleum ether (bp 40–60°C), the upper phase was collected and the solvent evaporated. After re-dissolving in acetone, the carotenoids were analyzed by HPLC on a 15 cm Nucleosil C18 column at 20°C with a mobile phase of acetonitrile/methanol /2-propanol (85:10:5). Absorbance at 450 nm and individual peaks were recorded with a Kontron DAD 440 photodiode array detector. Individual carotenoids were identified by comparing with authentic standards, their retention times, and absorbance spectra.

## Results

### Cloning and characterization of the maize *CYP97C19* gene

**Fig 2:**
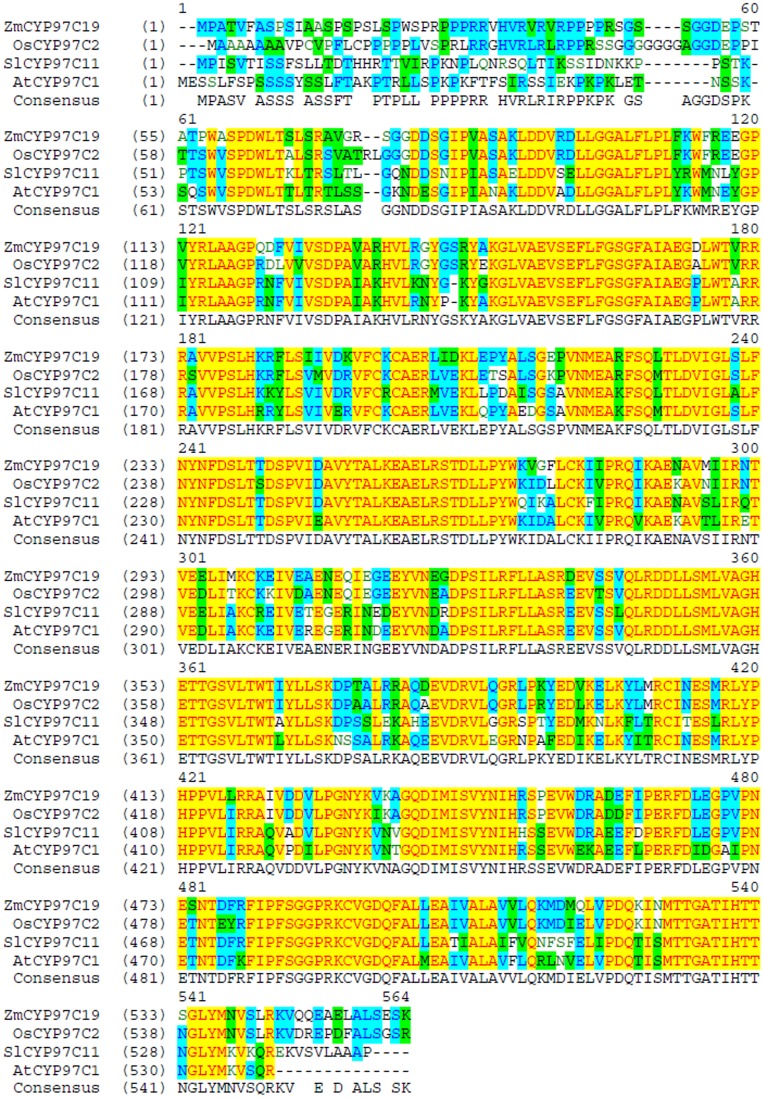
Multiple alignments of CYP97C protein sequences from maize (Zm, *Zea mays*; GenBank: GU130217), rice (Os, *Oryza sativa*; GenBank: AK065689), Arabidopsis (At, *Arabidopsis thaliana*; GenBank: NM_115173) and tomato (Sl, *Solanum lycopersicon*; GenBank: EU849604).

The maize *CYP97C19* cDNA encoding a full-length putative carotenoid ε-hydroxylase was amplified from the 25-DAP endosperm mRNA of maize inbred line B73 by RT-PCR (GenBank: GU130217). The full-length *ZmCYP97C19* cDNA encoded a 556-residue protein with a predicted molecular weight of 61.9 kDa. The chloroplast transit peptide prediction software ChloroP v1.1 indicated the presence of a putative 53-residue transit peptide. The ZmCYP97C19 amino acid sequence showed 88.6% similarity and 82.6% identity to rice CYP97C2, 80.8% similarity and 69.9% identity to *A*. *thaliana* CYP97C1, and 78.4% similarity and 68.7% identity to tomato CYP97C11 (Fig 2).

**Fig 3:**
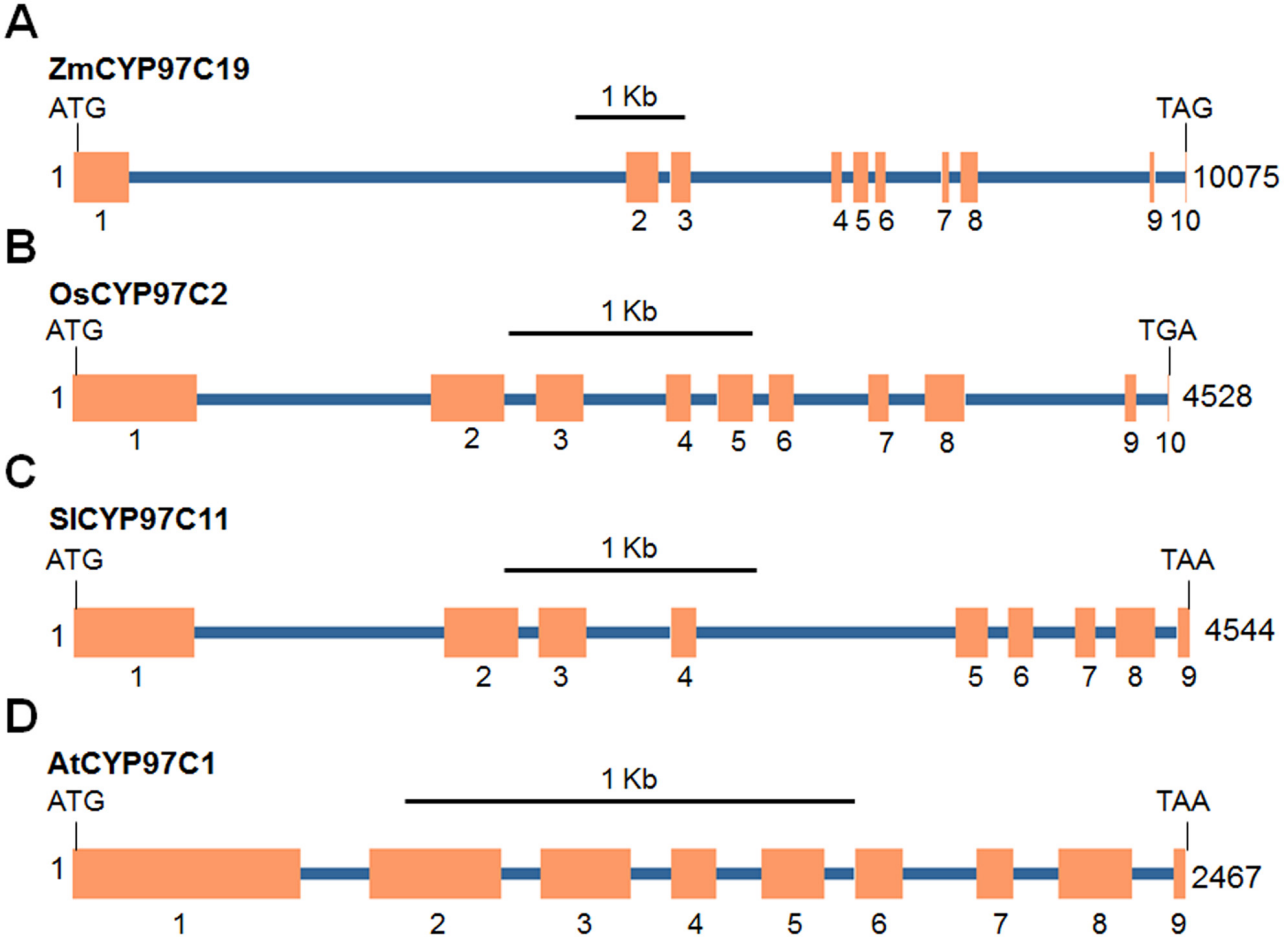
Gene structures for maize, rice, tomato and *A*. *thaliana* CYP97C homologs. Orange boxes represent exons. Blue lines represent introns. The lengths of DNA sequences are indicated on the right. Zm, *Zea mays* cDNA; GenBank: GU130217), rice (Os, *Oryza sativa* cDNA; GenBank: AK065689), Arabidopsis (At, *Arabidopsis thaliana* cDNA; GenBank: NM_115173), tomato (Sl, *Solanum lycopersicon* cDNA and genomic DNA; GenBank: EU849604 and EU849603).

The *ZmCYP97C19* cDNA sequence was used to screen MaizeGDB maize genomic resources to identify the corresponding gene. A single genomic sequence from chromosome 1 of the maize B73 genome matched the *ZmCYP97C19* cDNA sequence with 100% identity, suggesting that *ZmCYP97C19* is a single-copy gene (GenBank: AC177851). The *ZmCYP97C19* gene was found to have nine introns and ten exons (Fig 3) which is the same structure as the homologous rice gene *CYP97C2* [10]. In contrast, the homologous genes in *A*. *thaliana* (*CYP97C1*) and tomato (*CYP97C11*) have eight introns and nine exons [7,11].

### Screening and selection of transgenic *A*. *thaliana* plants

*A*. *thaliana lut1* mutant plants were transformed with the maize *CYP97C19* gene controlled by the constitutive CaMV 35S promoter, and self-pollination gave rise to T1 seeds that yielded hygromycin-resistant T1 plants. These plants were analyzed by genomic PCR to confirm the integrity of the *ZmCYP97C19* transgene using primers that annealed to the CaMV 35S promoter and *ZmCYP97C19* sequences. The complete *ZmCYP97C19* transgene was present in 15 T1 lines and leaves from these lines were used to determine carotenoid profiles by HPLC analysis. Three lines that accumulated the highest levels of lutein in the leaves were used for in depth analysis. T2 seedlings from these three self-pollinated T1 lines were selected on hygromycin, and rosette leaves were taken from these transgenic T2 plants as well as *lut1* mutant and wild-type controls. The leaves were used for HPLC analysis to determine the carotenoid profiles and DNA and RNA extraction for molecular characterization.

### Analysis of transgene integration

**Fig 4:**
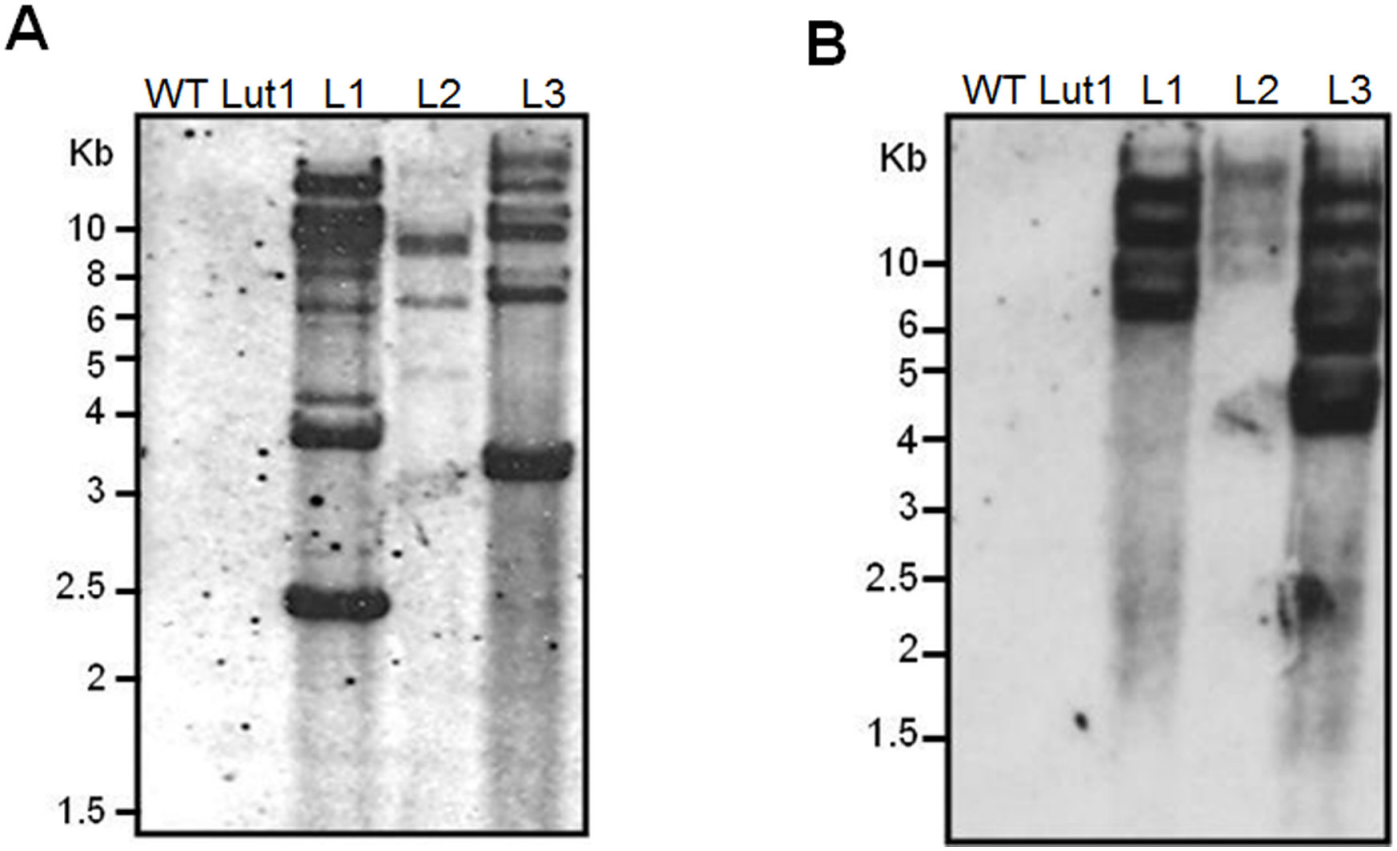
DNA blot analysis of *ZmCYP97C19* transgene in *A*. *thaliana* wild type, *lut1* mutant and three different transgenic lines transformed with *ZmCYP97C19* driven by the CaMV 35S promoter. Genomic DNA (10 μg) from rosette leaves was separately digested with *Eco*RI (A) and *Xba*I (B). The DNA blot was hybridized with a *ZmCYP97C19* gene probe. WT, wild-type (Col-0); Lut1, *lut1* mutant; L1, line 1; L2, line 2; L3, line 3.

The three transgenic T2 lines were compared by DNA blot analysis with wild-type and *lut1* mutant controls. The DNA was digested with *Eco*RI or *Xba*I and the blots were probed under high stringency conditions with a 1228-bp *ZmCYP97C19* DNA sequence lacking *Eco*RI and *Xba*I restriction sites. The results showed that the three transgenic lines had different hybridization band patterns indicating they were independent transformants, whereas the wild-type and *lut1* mutant controls did not show any hybridizing bands as expected (Fig 4). Multiple bands were visible on the DNA blots representing lines 1 and 3 regardless of which enzyme was used, indicating multiple copies of the transgene were present in the genome, whereas line 2 presented three bands with each of the enzymes, suggesting the presence of three transgene copies (Fig 4).

### Analysis of transgene expression

**Fig 5:**
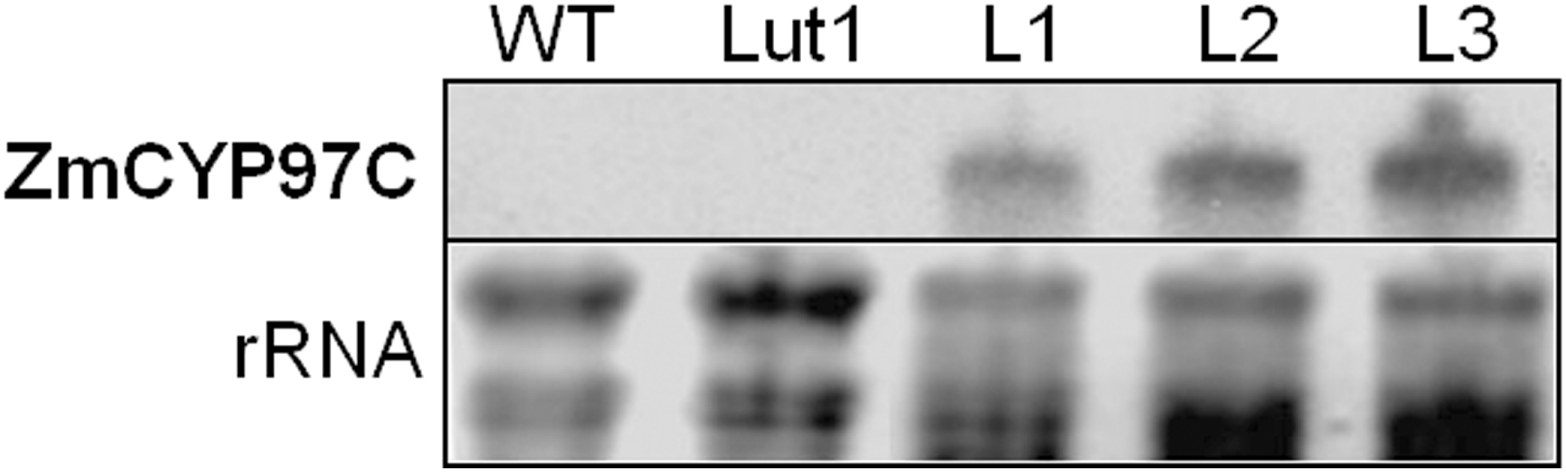
RNA blot analysis of maize *CYP97C19* transgene expression in *A*. *thaliana* wild-type, *lut1* mutant and three different transgenic lines transformed with *ZmCYP97C19* driven by the CaMV 35S promoter. Each lane was loaded with 20 μg total RNA from rosette leaves. Ribosomal RNA stained with ethidium bromide is shown as a loading control. The blot was hybridized with a *ZmCYP97C19* probe. WT, wild type (Col-0); Lut1, *lut1* mutant; L1, line 1; L2, line 2; L3, line 3.

Transgene expression was analyzed by mRNA blot, revealing that *ZmCYP97C19* mRNA was present in the rosette leaves of all three transgenic lines, whereas no mRNA was present in the controls (Fig 5). This confirmed that the transgene was intact and strongly expressed in all three transgenic lines.

### Analysis of carotenoid profiles

**Fig 6:**
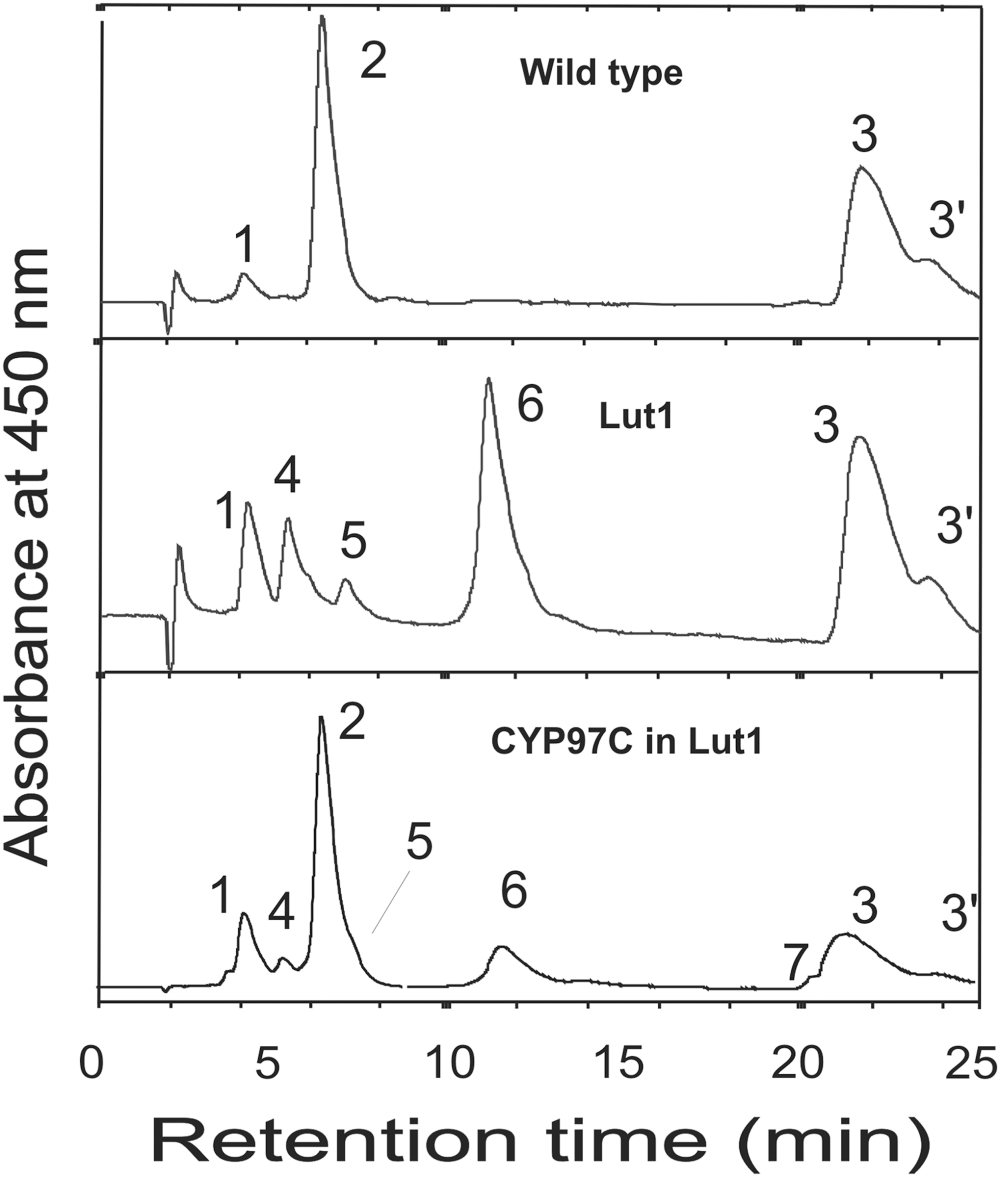
HPLC analysis of carotenoids in rosette leaves of *A*. *thaliana* wild-type, *lut1* mutant and transgenic lines transformed with *ZmCYP97C19* driven by the CaMV 35S promoter. Peak 1, violaxanthin; peak 2, lutein, peak 3, β-carotene; peak 3', β-carotene *cis* isomer; peak 4, antheraxanthin; peak 5, zeaxanthin; peak 6, zeinoxanthin; peak 7, α-carotene. Wild type, Col-0; Lut1, *lut1* mutant; CYP97C in Lut 1, transgenic lines.

The carotenoid composition of rosette leaves from the transgenic lines, wild-type plants and *lut1* mutants was determined by HPLC, and the results are summarized in Table 1. Lutein and β-carotene were the predominant carotenoids in wild-type leaves, whereas zeinoxanthin and β-carotene were the major carotenoids in the leaves of *lut1* mutant plants, but lutein was only present in trace amounts (Fig 6). In contrast, the expression of *ZmCYP97C19* in the *lut1* mutant background caused a significant increase in the lutein content (to 26.5%, 32.2% and 49.6% of total carotenoids in transgenic lines 1, 2 and 3, respectively). The lutein appeared to be derived from zeinoxanthin, because the abundance of this carotenoid was reduced from 36.5% in the *lut1* mutant to 15.5%, 14.7% and 7.3% in transgenic lines 1, 2 and 3, respectively (Fig 6 and Table 1). *ZmCYP97C19* therefore appears to encode a functional carotenoid ε-hydroxylase, which catalyzes the conversion of zeinoxanthin to lutein by adding a hydroxyl group at the 3′ position of the ε-ring (Fig 1). The transgenic lines also accumulated higher levelsof violaxanthin than the *lut1* mutant, this being the major β,β-xanthophyll, but lower levels of β-carotene, zeaxanthin and antheraxanthin (Table 1).

**Table 1:** Abundance of individual carotenoids in *Arabidopsis thaliana* leaves (%) and the total carotenoid content (μg/g dry weight).

	Nx	Viox	Anx	Lut	Zeax	HOaC	aCar	bCar	Total Car
**WT**	**1.1±0.4**	**5.7±2.6**	**1.6±1.0**	**54.9±5.4**	**tr**	**nd**	**tr**	**35.8±8.3**	**1271.3±385.5**
***lut1***	**1.1±0.3**	**7.5±0.8**	**10.1±1.1**	**tr**	**3.8±0.3**	**36.5±2.8**	**tr**	**41.0±3.1**	**719.3±208.9**
**Line 1**	**1.6±0.8**	**17.2±4.6**	**6.1±1.4**	**26.5±8.3**	**tr**	**15.7±5.3**	**tr**	**29.1±2.5**	**1251.7±116.2**
**Line 2**	**1.2±0.3**	**20.0±4.2**	**4.8±0.9**	**32.2±4.3**	**2.6±2.0**	**14.1±0.8**	**tr**	**25.2±2.3**	**923.7±96.5**
**Line 3**	**1.3±0.5**	**13.4±1.1**	**4.0±0.4**	**49.6±2.6**	**tr**	**7.3±1.0**	**tr**	**22.6±2.3**	**913.0±202.7**

Values are mean ± standard deviation of at least three replicates. Abbreviations: Nx, neoxanthin; Viox, violaxanthin; Anx, antheraxanthin; Lut, lutein; Zeax, zeaxanthin; HOaC, zeinoxanthin; aCar, α-carotene; bCar, β-carotene; Total Car, total carotenoids; nd, not detected; tr, trace, less than 0.1%; WT, wild type.

## Discussion

The catalytic activities of different carotenogenic enzymes determine the abundance and composition of carotenoids in maize endosperm. Phytoene synthase (PSY) is a major rate-limiting step in the carotenoid biosynthesis pathway. Multiple isozymes of PSY regulate carotenogenesis in a tissue-specific manner in maize and rice [26,27] and fluctuating levels of the enzymes exert quantitative effects on the carotenoid content, as demonstrated in transgenic canola [28], tomato [29] and maize [30]. Other enzymes are responsible for the carotenoid profile. For example, lycopene ε-cyclase (LYCE) plays a key role by competing with lycopene β-cyclase (LYCB) to regulate the formation of α-carotene and its derivatives in maize endosperm [31].

Two classes of structurally-unrelated enzymes catalyze the hydroxylation of α- and β-ionone rings in higher plants. These are the CYP97-type heme-containing cytochrome P450 hydroxylases [7,8] and the ferredoxin-dependent BCH-type non-heme di-iron hydroxylases [12,13,32]. These enzyme classes have overlapping substrate specificities but *in vivo* analysis has shown that BCH isozymes are predominantly responsible for the synthesis of β,β-xanthophylls, i.e. they have limited activity towards the ε-ring of α-carotene but significant activity towards the β-ring with exception of the maize crtRB3 (also known as BCH1) that affects the accumulation of α-carotene [33].

In contrast, the CYP97 enzymes have evolved to function preferentially the synthesis of α-xanthophylls and show substantial divergence in their preferences for *in vivo* substrates. Maize BCH2 (also known as HYD3 and CrtRB1) is developmentally regulated but preferentially expressed in the endosperm, where it governs the critical steps in the conversion of β-carotene to zeaxanthin via β-cryptoxanthin [15,34–37]. Hypomorphic alleles therefore cause the accumulation of β-carotene [34,35].

Many BCH-type β-carotene hydroxylases from higher plants have been extensively characterized, allowing their use in rational metabolic engineering strategies [5,38,39]. However, only the *A*. *thaliana*, rice and tomato carotenoid ε-hydroxylases have received similar attention [7,9–11]. *A*. *thaliana* CYP97C1 shows high activity towards the α-carotene ε-ring and moderate activity toward the β-ring, but minimal activity toward the β-rings of β-carotene [7,9]. In contrast, rice CYP97C2 shows weak ε-ring hydroxylase activity and no β-ring hydroxylase activity in *E*. *coli* cells accumulating ε-carotene (not an *in vivo* substrate) or β-carotene [10]. Tomato CYP97C11 only shows activity towards the ε-ring of α-carotene [11]. The preferred pathway for lutein synthesis in *A*. *thaliana*, rice and tomato is through the sequential action of CYP97A and CYP97C [9,11,40]. CYP97A converts α-carotene to zeinoxanthin, which is in turn hydroxylated by CYP97C to form lutein. In tomato, hydroxylation of the ε-ring of zeinoxanthin by CYP97C11 appears to be the most critical step in lutein synthesis because the activity of this enzyme cannot be replaced by CYP97A29 or by either of the tomato BCH-type carotenoid hydroxylases. The hydroxylation of α-carotene to lutein in tomato is therefore mediated by the β-hydroxylation of α-carotene to zeinoxanthin catalyzed by CYP97A29 followed by the ε-ring hydroxylation of zeinoxanthin to lutein by CYP97C11 [11]. The first step can be partially complemented by CRTR-B1 (BCH1), CRTR-B2 (BCH2) or CYP97C11, but the ε-ring of zeinoxanthin can only be hydroxylated by CYP97C11 [11]. The constitutive overexpression of the tomato carotenoid ε-hydroxylase CYP97C11 in transgenic tobacco significantly increased the amount of lutein in the leaves and alleviated the photo-inhibition and photo-oxidation caused by chilling stress [17].

The cDNA encoding the putative carotenoid ε-hydroxylase CYP97C19 was isolated from maize endosperm and constitutively overexpressed in the *A*. *thaliana lut1* knockout mutant, which has the low-lutein *cyp97c1* mutant phenotype. This was confirmed by the analysis of carotenoid pigments in wild-type and *lut1* mutant plants, which showed carotenoid profiles consistent with previous results [9]. The lutein levels in transgenic *A*. *thaliana* plants overexpressing *ZmCYP97C19* were much higher than in the untransformed *lut1* mutant although not as high as wild-type levels (Table 1). Furthermore, the high levels of zeinoxanthin in the *lut1* mutant were reduced in the transgenic lines, confirming that ZmCYP97C19 is an ε-hydroxylase that can use zeinoxanthin as a substrate. However, we did not detect α-cryptoxanthin, the α-carotene derivative hydroxylated at position 3 of the ε-ring, in either the *lut1* mutant or the transgenic lines, whereas trace amounts were present in wild-type leaves (Table 1 and Fig 6). This suggests that α-carotene may not be a preferred substrate for ZmCYP97C19, or that any α-cryptoxanthin thus formed is efficiently converted to lutein by the endogenous β-ionone ring hydroxylase.

The carotenoid content and composition of maize endosperm varies substantially between varieties reflecting different patterns of carotenogenic gene expression [31,41]. The expression of *PSY1*, *HYD3* (*BCH2*) and *CYP97C* has recently been evaluated in 22 different maize landraces [41]. High levels of *ZmCYP97C* expression levels or a low *HYD3/CYP97C* expression ratio correlated positively with high lutein levels, which is consistent with our finding that ZmCYP97C is needed to produce lutein. In contrast, high levels of *HYD3* (*BCH2*) expression or a high *HYD3/CYP97C* expression ratio correlated positively with high zeaxanthin levels [41]. The *ZmCYP97C19* mRNA levels remained constant throughout endosperm development in the white maize inbred variety M37W [42].

The functional analysis of enzymes in crops is necessary for the development of targeted metabolic interventions. In this context, ZmCYP97C19 appears to be important because of its key role in lutein biosynthesis and therefore its potential application in cereals for lutein biofortification. Lutein is increasingly regarded as an essential nutrient because of its proposed role in maintaining vision and preventing age-related maculopathy [3]. Lutein is also valuable in the food, feed and nutraceutical markets as an additive and health-promoting natural product [4,43]. A better understanding of the regulation of lutein synthesis in plants is therefore likely to be valuable for human and animal health and in the commercial development of carotenoid-based supplements.
